# Deep Brain Stimulation during Pregnancy and Delivery: Experience from a Series of “DBS Babies”

**DOI:** 10.3389/fneur.2015.00191

**Published:** 2015-09-01

**Authors:** Emma Scelzo, Jan H. Mehrkens, Kai Bötzel, Paul Krack, Alexandre Mendes, Stéphan Chabardès, Mircea Polosan, Eric Seigneuret, Elena Moro, Valerie Fraix

**Affiliations:** ^1^Department of Neurology, Policlinico San Donato, Milan University, Milan, Italy; ^2^Clinical Center for Neurotechnology, Neurostimulation and Movement Disorders, Fondazione IRCCS Ca’ Granda – Ospedale Maggiore di Milano, Milan, Italy; ^3^Department of Neurology, Grenoble University Hospital, Grenoble, France; ^4^Department of Neurosurgery, Ludwig-Maximilians-University, Munich, Germany; ^5^Department of Neurology, Ludwig-Maximilians-University, Munich, Germany; ^6^Grenoble Institute of Neurosciences, INSERM U836, Joseph Fourier University, Grenoble, France; ^7^Department of Neurology, Porto University Hospital, Porto, Portugal; ^8^Department of Neurosurgery, Grenoble University Hospital, Grenoble, France; ^9^Department of Neuropsychiatry, Grenoble University Hospital, Grenoble, France

**Keywords:** deep brain stimulation, dystonia, obsessive compulsive disorder, Parkinson disease, pregnancy, teratogenicity, Tourette’s syndrome

## Abstract

**Introduction:**

Deep brain stimulation (DBS) is widely used to improve quality of life in movement disorders (MD) and psychiatric diseases. Even though the ability to have children has a big impact on patients’ life, only a few studies describe the role of DBS in pregnancy.

**Objective:**

To describe risks and management of women treated by DBS for disabling MD or psychiatric diseases during pregnancy and delivery.

**Methods:**

We report a retrospective case series of women, followed in two DBS centers, who became pregnant and went on to give birth to a child while suffering from disabling MD or psychiatric diseases [Parkinson’s disease, dystonia, Tourette’s syndrome (TS), Obsessive Compulsive Disorder (OCD)] treated by DBS. Clinical status, complications and management before, during, and after pregnancy are reported. Two illustrative cases are described in greater detail.

**Results:**

DBS improved motor and behavioral disorders in all patients and allowed reduction in, or even total interruption of disease-specific medication during pregnancy. With the exception of the spontaneous early abortion of one fetus in a twin pregnancy, all pregnancies were uneventful in terms of obstetric and pediatric management. DBS parameters were adjusted in five patients in order to limit clinical worsening during pregnancy. Implanted material limited breast-feeding in one patient because of local pain at submammal stimulator site and led to local discomfort related to stretching of the cable with increasing belly size in another patient whose stimulator was implanted in the abdominal wall.

**Conclusion:**

Not only is it safe for young women with MD, TS and OCD who have a DBS-System implanted to become pregnant and give birth to a baby but DBS seems to be the key to becoming pregnant, having children, and thus greatly improves quality of life.

## Introduction

Deep brain stimulation (DBS) is considered as an effective treatment in patients with advanced Parkinson’s Disease (PD) (1), and medically poorly responsive movement disorders (MD) and psychiatric diseases such as dystonia, Tourette’s syndrome (TS) and obsessive compulsive disorder (OCD) (2–4). Recent studies have shown that its early application improves quality of life, and prevents psychosocial and functional impairment (5–7).

The ability to have children and a good family life has a large impact on quality of life, especially in young patients (8, 9). Because caring for a child requires good health, having a disabling progressive disease often prevents young women from becoming pregnant. A number of authors (10–13) have, moreover, reported worsening of MD and OCD during pregnancy, exacerbated by a reduction in medical treatment to avoid possible teratogenicity (14).

Deep brain stimulation improves patients’ clinical status and allows reduction of medical treatment (1–3). Its non-systemic action could, moreover, improve symptoms during pregnancy without affecting fetal development.

We report a case series of patients affected by disabling neurological and psychiatric diseases who became pregnant while being treated by DBS, and focus on patients’ psychomotor status and treatment management during pregnancy and delivery.

## Methods

We retrospectively included all patients affected by disabling MD and psychiatric diseases operated on in our centers who became pregnant while being treated by DBS. We collected demographic characteristics and histories of disease and pregnancy from patients’ records. We assessed the clinical status before (OFF and ON medication, after a supraliminal levodopa dose for PD) and 1 year after surgery (OFF and ON DBS, OFF and ON medication, after a supraliminar levodopa dose for PD) and before, during, and after pregnancy (ON DBS, chronic medical treatment), using the Unified Parkinson’s Disease Rating Scale (UPDRS) motor score (part III) in PD; the Burke–Fahn–Marsden Dystonia Rating Scale (BFMDRS) [motor score (MS) and disability score (DS)] in dystonia and Toronto Western Spasmodic Torticollis Rating Scale (TWSTRS) in cervical dystonia (patient 8); the Yale Global Tic Severity Scale (YGTSS) in TS and the Yale-Brown Obsessive Compulsive Scale (YBOCS) in OCD. We then collected the non-available information through phone interviews.

*The participants who were included in the study had given their written informed consent according to the Center’s Review board guidelines and the Declaration of Helsinki. All participants gave their informed consent to report their personal and medical data in this clinical cases study. Our Centers did not require ethical board approval for the treatments presented in the manuscript and to publish retrospective and anonymous data, with no statistical result*.

## Patient Characteristics before and after DBS

Eleven patients (three PD, five dystonic, two TS and one OCD patients) were included between 2010 and 2014. Six patients were operated on at the Grenoble University Hospital and five at the Munich Ludwig-Maximilians University.

Before DBS, all PD patients had developed severe motor fluctuations and dopamine dysregulation syndromes with severe behavioral disorders resulting in fateful outcomes such as marital breakdown, loss of child custody, depression, and suicidal ideation. All dystonic, TS, and OCD patients had drug resistant symptoms or drug side effects and very low levels of social functioning, avoiding contact. The mean age ±SD at DBS (bilateral subthalamic nucleus for PD, OCD and one dystonic patient; bilateral globus pallidus internus for dystonia and TS) was 27.5 ± 7.0 years. Pulse generators (Medtronic: Kinetra Dual channel in seven patients, Soletra in one patient, Activa PC dual-channel in three patients) were implanted in subclavicular (eight patients) or abdominal (three patients) sites.

Deep brain stimulation induced a clear symptomatic improvement in all patients, and this was confirmed by scores on 12-month post-DBS clinical assessment scales (see Table 1). PD patients experienced a reduction in their motor symptoms, fluctuations, and behavioral disorders. In one PD patient (patient 2), minimal left side rigidity, akinesia and apathy persisted 1 year after DBS. Functional ability improved in all dystonic patients. Because of progressive worsening of dystonia almost 1 year after surgery, a second globus pallidus internus DBS was performed on patient 7 6 years later. TS patients experienced tic reduction, and behavioral improvement was noted in TS and OCD patients.

**Table 1:** **BS effects on clinical assessment scales and treatment management**.

Patients	Disease	Scale scores						Medical treatment	
		Before DBS		After DBS				Before DBS	After DBS
		Off Med	On Med	Off Med Off Stim	Off Med On Stim	On Med Off Stim	On Med On Stim	
1	Parkin mutation PD	42/108	19/108	43/108	28/108	15/108	9/108	L-dopa-carbidopa-entacapone 350 mg	Pramipexole CR 1.05 mg
								Pramipexole 0.9 mg	Rasagiline 1 mg
2	Parkin mutation PD	29/108	5/108	39/108	24/108	27/108	11/108	L-dopa-benserazide 300 mg	Piribedil 300 mg
								Ropinirole 6 mg	
3	Parkin mutation PD	30/108	4/108	35/108	19/108	8/108	4/108	L-dopa-benserazide 600 mg	Bromocriptine 15 mg
								Ropinirole CR 8 mg	L-carbidopa CR 200 mg
4	Post-anoxic dystonia	NA	MS 24/120	NA	NA	NA	MS 5/120	Clonazepam 3 mg	Clonazepam 0.3 mg
			DS 6/29				DS 2/29	
5	DYT 1 Dystonia	MS 32/120	NA	NA	MS 6/120	NA	NA	No	No
		DS 14/29			DS 4/29	
6	DYT 1 Dystonia	MS 40/120	NA	NA	MS 10/120	NA	NA	No	No
		DS 16/29			DS 4/29	
7^a^	DYT 1 Dystonia	NA	MS 26/120	NA	MS 1/120	NA	NA	Trihexyphenidyl 15 mg	No
			DS 9/29		DS 0/29			
								Baclofen 100 mg	
8	Primary segmental dystonia	22/87	NA	NA	2/87	NA	NA	No	No
9	TS	NA	89/100	NA	32/100	NA	NA	Sertraline 100 mg	No
								Haloperidol 5 mg	
								Valproic acid 600 mg	
10	TS	NA	91/100	NA	30/100	NA	NA	Sertraline 150 mg	No
								Olanzapin 5 mg	
11	OCD	NA	22/40	NA	12/40	NA	NA	Escitalopram 30 mg	No

*Clinical assessment scale scores and medical treatment before (Off and On medication, after a supraliminar levodopa dose for PD) and 1 year after DBS (Off and On DBS, Off and On Medication, after a supraliminar levodopa dose for PD) are listed. We reported the Unified Parkinson’s Disease Rating Scale (UPDRS) motor score (part III) in PD; the Burke–Fahn–Marsden Dystonia Rating Scale (BFMDRS) [motor score (MS) and disability score (DS)] in dystonia and Toronto Western Spasmodic Torticollis Rating Scale (TWSTRS) in cervical dystonia (patient 8); the Yale Global Tic Severity Scale (YGTSS) in TS and the Yale-Brown Obsessive Compulsive Scale (YBOCS) in OCD*.

*NA, not available; Med, medication; Stim, stimulation*.

*^a^First DBS*.

Deep brain stimulation permitted a reduction in medical treatment (see Table 1). *After DBS, the levodopa equivalent daily dose was reduced from 555 to 205 mg/day in patient 1 and from 760 to 325 mg/day in patient 3 and was increased from 420 to 500 mg/day in patient 2* (15).

One dystonic patient (patient 7), the OCD and the TS patients stopped their treatment, while another dystonic patient (patient 4) reduced it by 90%. The others had no treatment before or after surgery.

## Patients’ Pregnancies

Prior to DBS, two PD patients had had one and two elective abortions, respectively, either because of a fear of treatment’s effects on the fetus or of their behavioral dopamine dysregulation syndromes. One patient had had two spontaneous abortions for unknown reasons (the first when taking levodopa, the second when taking rasagiline, trihexyphenidyl, and levodopa). Two PD patients and the OCD patient had had two and three healthy pregnancies, respectively.

After DBS, ten patients had one pregnancy, and one patient (patient 9) had two. The mean delay between DBS and pregnancy was 4.7 ± 3.4 years (mean ± SD).

During pregnancy under DBS, four patients reported no change in their clinical status, two reported clinical improvement and five mild clinical worsening (patient 9 in both pregnancies) with an increase in severity of fatigue, motor fluctuations, dystonia or tics.

Apart from two PD patients (one patient from the 6 month of pregnancy only), patients took no medication during pregnancy (see Table 2). DBS was adjusted to improve symptoms in five patients (see Table 2).

**Table 2:** **Medical treatment and DBS parameters during pregnancy**.

Patients’ pregnancies	Medical treatment			DBS parameter changes during pregnancy
	Before pregnancy	During pregnancy	After pregnancy	
1	Pramipexole CR 1.05 mg	No	Pramipexole CR 1.05 mg rasagiline 1 mg	No
	Rasagiline 1 mg	
2	Ropinirole 14 mg	Levodopa 200 mg	Ropinirole 10 mg	No
3^a^	Rasagiline 1 mg ropinirole 2 mg trihexyphenidyl 150 mg	Rasagiline 1 mg ropinirole 2 mg trihexyphenidyl 150 mg	Rasagiline 1 mg ropinirole 2 mg trihexyphenidyl 150 mg	No
4	Clonazepam 0.3 mg	No	No	Yes, voltage decrease
5	No	No	No	No
6	No	No	No	No
7	Baclofen 100 mg	No	No	Yes, voltage increase
8	No	No	No	No
9 (1)	No	No	No	Yes, voltage increase
9 (2)	No	No	No	Yes, voltage increase
10	No	No	No	Yes, voltage increase
11	No	No	No	Yes, voltage increase

*The table shows medical treatment and DBS parameter management in each patient’s pregnancy*.

*^a^On medication from the 6 month of pregnancy. Patient 9 (1, 2): first and second pregnancy*.

All deliveries occurred at full term. Three patients had vaginal births while the others had cesarean sections (C-section) (one due to the baby’s abnormal position, one to avoid clinical impairment at delivery, one due to previous C-section, and the others because of the unknown risks of delivery on implanted devices). *The C-section was performed according to the own protocol of each obstetrical center*.

All babies were healthy and had a normal course of development. One patient experienced spontaneous abortion of one fetus in the first weeks of a twin pregnancy. Her second baby was healthy on delivery.

Eight patients breastfed their babies. PD patients on oral treatment, however, did not, because of the unknown toxicity of medication. One dystonic patient was unable to breastfeed because of pain at the submammary stimulator site.

After delivery, the conditions of two patients worsened temporarily. Another patient experienced chronic progressive worsening of her dystonia. This improved after electrode implantation in the subthalamic nucleus. Conversely, TS patients reported an improvement. While medical treatment was reintroduced in PD patients, dystonic patients who had discontinued treatment before pregnancy remained OFF treatment. Stimulation parameters were set at pre-pregnancy levels in TS patients 3 months after delivery. They were changed for two patients whose condition worsened after delivery.

The pulse generator battery required replacement a few days after delivery in two patients and during pregnancy in one. One patient complained of discomfort accompanied by a sensation of “tension” along the extension cable (abdominal stimulator site) during pregnancy.

At the final follow-up (19.0 ± 14.8 months after delivery; mean ± SD) all patients were independent and able to take care of their babies.

## Illustrative Clinical Cases

### First clinical case: A PD patient

This case illustrates the role of DBS on motor symptoms and social adjustment in PD with motor and non-motor levodopa complications. Patient 1 developed PD due to Parkin mutation at the age of 19. A few years after the onset of PD, she married and had two pregnancies (two daughters, one healthy and the other suffering from a severe atrial defect). Like most young PD patients, she progressively developed motor complications such as painful OFF dystonia and dyskinesia. By the time she was 34, these had become disabling. The dopamine agonist daily dosage was increased to improve motor fluctuations and, when taking pramipexole 2.1 mg/day, she developed severe dopamine dysregulation syndrome with hypersexuality, compulsive shopping and nocturnal hyperactivity. She became pregnant for a third time but aborted quickly because of the fear of possible teratogenic effects of her treatment. The family equilibrium was upset, she divorced and lost custody of the children. Since her quality of life was progressively worsening because of her motor fluctuations and behavioral disorders, she underwent subthalamic nucleus DBS (Medtronic DBS 3389 electrodes, pulse generator Kinetra) in 2009 at the age of 35. DBS greatly improved her motor symptoms (UPDRS III fell from 19/108 to 9/108 (−52.6%) and fluctuations and allowed for a reduction in dopaminergic treatment, with a subsequent improvement in her behavior. One year after surgery, she was allowed to bring her daughters back home and to care for them on her own. Two years after surgery, she met a new partner and decided to have another child. During pregnancy, she was able to stop dopaminergic treatment without any complication or worsening of symptoms. Delivery occurred at full term by C-section because of the baby’s abnormal position. She was able to breastfeed her baby safely. Some weeks after delivery she noticed an occasional worsening of right leg dystonia and impairment in walking, but her UPDRS motor score remained unchanged. She now lives alone during the week with her three daughters, as her partner lives and works away from home.

### Second clinical case: A TS patient

This case illustrates the effect of DBS on psychosocial competence in patients with neuropsychiatric diseases. Patient 9 developed TS at the age of 6. Her symptoms included severe coprolalia, consisting mainly of politically incorrect words, and self-injurious behavior such as burning her skin with an electric iron, cutting her hair, and painting on her skin. Her level of social functioning was extremely low – she lived only with her partner and walked her dog at night to avoid meeting other people. Acute 12-fold electroconvulsive therapy had suppressed tics and was followed by 8-monthly maintenance treatment, which had to be discontinued due to non-convulsive status epilepticus. As a result, posteroventrolateral globus pallidus internus DBS (Medtronic DBS 3387 electrodes, pulse generator Kinetra) was performed at the age of 28. DBS reduced almost all symptoms within 12 months [(Clinical Global Impression scale: from 6 to 3/7, TS Global Scale: from 71.3/100 to 14/100 (−80%), YGTSS: from 89/100 to 32/100 (−64%)] allowing her to return to part time work in her previous occupation as an administrative assistant. Postoperatively, and having discontinued any TS-specific medication, she married, became pregnant and gave birth to a healthy boy. In the 34th week of pregnancy, the pulse generator battery had almost run out and required replacement; the patient’s obstetrician/gynecologist had to put this on stand-by in case the patient required an emergency C-section. No complications occurred during battery replacement. With her tics almost completely suppressed and able to do without medication, the patient became pregnant for a second time and gave birth to another healthy boy (16).

## Discussion

In our case series, MD patients had disabling motor symptoms. All PD, TS, and OCD patients had developed severe behavioral disorders resulting in fateful outcomes such as marital breakdown, loss of child custody, depression, and suicidal ideation or avoidance of social contact, which all compromised the possibility of having children.

Deep brain stimulation induced clinical and behavioral improvement and permitted the reduction of medical treatment, in line with the literature (1–4). It therefore contributed to the possibility of having and rearing children, and limited fetal exposure to medication.

With the exception of the spontaneous abortion of one fetus within the first weeks of a twin pregnancy, all pregnancies were uneventful. As previously reported, our study revealed conflicting data on patients’ clinical status during pregnancy (10–13, 17, 18). Most women were, however, able to go to term with no difficulty. Some case reports, concerning mainly PD, have shown possible disease worsening during pregnancy or in the postpartum period (10–12) while other reports claim to have noted no change or even improvement (17, 18). Few uneventful pregnancies have been described in women with dystonia treated by DBS (19, 20).

In our study, DBS adjustments limited clinical worsening in five patients and allowed nine out of eleven women to take their pregnancies to term without treatment. Uneventful pregnancies have been described in MD and OCD patients on medical treatment (9–14, 17, 18, 21–24). However, most medications used in MD and OCD are considered pregnancy class C due to the lack of evidence about their impact on fetal development and teratogenic risk (9, 23, 24). Women are, therefore, often forced to reduce or change their treatment, with ensuing clinical worsening (14, 25). Our data support the idea that, because of its non-systemic action, DBS could be a better way than medical treatment alone of controlling patients’ symptoms during pregnancy.

A growing number of women of childbearing-age have a DBS-system. To our knowledge, our case series is the largest available and we did not encounter any major adverse effects related to DBS. Two patients had routine battery replacements, a few days after delivery and one required battery replacement under local anesthetic during pregnancy. These cases underline the need to anticipate battery replacement in planned pregnancies in order to avoid surgery and possible worsening of symptoms during pregnancy. One patient was unable to breastfeed because of pain at the neurostimulator site (submammary site) and another one experienced discomfort accompanied by a sensation of tension in the abdominal skin along the extension cable (abdominal site) toward the end of pregnancy when increasing belly size stretched the cable. These side effects support the need to consider different device-related options in childbearing-age patients who wish to become pregnant. C-sections were performed on five patients because of the lack of evidence regarding natural birth under DBS. We did not report any complication during natural birth in our patients. This supports the safety of DBS during pregnancy and at delivery for both mother and child if the underlying disease is well controlled by DBS.

Our report had several limits. First, because of its retrospective nature, data collection was not complete and scale scores were not available for all patients. This limited the possibility of objectively confirming clinical changes in all clinical assessments. We were, in addition, unable to quantify daily life activities and quality of life changes. Second, even though it is the largest series available, our sample remains small and prospective and larger studies are needed.

In conclusion, because of the effectiveness of DBS on psychomotor status and treatment reduction, our report suggests it has a potential role in the management of young women suffering from disabling MD and OCD who wish to become pregnant. It also stressed the need to define strategies to prevent and control any worsening of patients’ condition during pregnancy, and to consider device-related options, such as choice of stimulator type (mono or *dual-channel*, primary cell or rechargable) and implantation site (subclavicular and abdominal) in women who plan to become pregnant.

## Author Contributions

ES, JM, VF, PK, and KB made a substantial contribution to the conception and design of the work. ES, JM, VF, AM, and MP contributed to collecting patients’ data and revising the drafting work. *ES and JM contributed to the manuscript redaction at the same level. All the authors (ES, JM, KB, PK, AM, VF, SC, MP, ES, EM, and VF) made a substantial contribution to the interpretation of the results, revised the draft and gave their final approval of the version to be published.

## Conflict of Interest Statement

The research was conducted in the absence of any commercial or financial relationships that could be construed as a potential conflict of interest.

## Abbreviations

BFMDRS, Burke–Fahn–Marsden Dystonia Rating Scale; C-section, cesarean section; DBS, deep brain stimulation; MD, movement disorder; OCD, obsessive-compulsive disorder; PD, Parkinson disease; TS, Tourette’s syndrome; TWSTRS, Toronto Western Spasmodic Torticollis Rating Scale; UPDRS, Unified Parkinson’s Disease Rating Scale; YBOCS, Yale-Brown Obsessive Compulsive Scale; YGTSS, Yale Global Tic Severity Scale.
